# Evidence that intrinsic brain connectivity moderates the association between gait and neurocognitive function in people living with HIV

**DOI:** 10.1007/s13365-026-01348-0

**Published:** 2026-10-13

**Authors:** Jonathan C. Ipser, Samantha Smuts, Hetta Gouse, Carla Freeman, Dan J. Stein, John Joska

**Affiliations:** 1 Department of Psychiatry and Mental Health, Neuroscience Institute, University of Cape Town, Rm 23, Anzio Road, Observatory, Cape Town, 7925 South Africa; 2 Te Toka Tumai, Te Whatu Ora, Auckland, New Zealand; 3 MRC Unit on Risk & Resilience in Mental Disorders, University of Cape Town, Cape Town, South Africa; 4 Department of Psychiatry and Mental Health, HIV Mental Health Research Unit, University of Cape Town, Cape Town, South Africa

**Keywords:** HIV, Gait speed, Neurocognitive function, Functional connectivity, Frontoparietal network, Executive function

## Abstract

Globally, a substantial proportion of the approximately 40 million people living with HIV (PLWH) experience some degree of HIV-associated brain injury (HABI), which is often linked to accentuated or accelerated aging in this clinical population. We set out to test the hypothesis that gait speed is associated with cognitive deficits in middle-aged PLWH in a low-middle income (LMIC) setting, and that this is comparable to what is seen in much older healthy adults. In addition, we sought to identify, as a potential marker of HABI, intrinsic functional brain connectivity abnormalities that moderate the association between gait and cognitive function in PLWH. Thirty-nine participants (17 HIV + and 22 HIV-, mean age = 39 +-8.99 years) were recruited from a community health centre in Cape Town. Bivariate and multiple linear regression models compared PLWH and controls with respect to associations between gait speed and neurocognitive domain scores, with relations between these factors and functional connectivity of seven resting state networks assessed within a multivariate regression modelling framework. We found that slower gait speed in PLWH was associated with lower global neurocognitive scores (t = -3.12; *p* < 0.008), and that this association was replicated in a large public domain cohort of healthy older adults. Moreover, intrinsic connectivity of the frontoparietal network in PLWH was a significant predictor of both gait speed and executive function, a finding that may implicate impaired compensatory mechanisms in this patient population. Our findings underscore the potential utility of gait tests in screening for HABI in PLWH in low resourced settings.

**Supplementary Information:**

The online version contains supplementary material available at https://doi.org/10.1007/s13365-026-01348-0.

## Introduction

According to the World Health Organization, roughly 40.8 million people worldwide were living with human immunodeficiency virus (HIV) at the end of 2024 (WHO, 2026). The profile of neurocognitive impairment (NCI) in people living with HIV (PLWH) has changed in the antiretroviral therapy (ART) era due to improved viral suppression and increased life expectancy (Frescura et al. 2022; van Sighem et al. 2010). Decrements in cognitive function observed in an aging patient population presents challenges to the successful treatment of HIV infection, as well as substantially impacting quality of life and everyday functioning (Alford et al. 2022; Mayo et al. 2020). In addition, PLWH may face cognitive decline from a multitude of other causes, including pre-treatment damage from central nervous system infection with HIV, opportunistic infections, side effects of ART, and non-HIV comorbidities (Alford and Vera 2018; Chan and Valcour 2021). The International HIV-Cognition Working Group recommends considering HIV-associated brain injury (HABI), which requires evidence of brain injury alongside clinical and cognitive assessments, as part of a broad differential for identifying the cause of NCI in PLWH (Nightingale et al. 2023). These recommendations reflect the recognition that linking brain mechanisms to cognitive test performance may reduce the high false positive rates for NCI resulting from diversity in educational, cultural, and socioeconomic factors (Underwood et al. 2018; Gisslen et al. 2011).

Roughly two decades ago, Robertson and colleagues reported significant correlations between walking time and testing scores on neurological and neurocognitive evaluations in PLWH (Robertson et al. 2006), a finding that was subsequently independently corroborated in older adult PLWH by a number of other research teams (Masters et al. 2019; Bourgeois et al. 2020). On the basis of these findings, it has been argued that slower gait speed may be an important clinical sign of NCI in PLWH (Derry et al. 2020). Being able to use gait speed to screen for NCI, and more specifically, HABI, in PLWH would be particularly useful in low and middle-income country (LMIC) settings, given the onerous and resource intensive task of assessing neurocognitive function in these patients. The timed gait test has particular promise as a screening tool as it is a quick, inexpensive, and non-invasive assessment that requires minimal training and no specialized equipment.

Greater convergence of neurocognitive and walking ability is typically observed in older healthy individuals and patient groups compared to younger healthy controls (Doi et al. 2014; Buracchio et al. 2010). However, there is little research that directly compare groups that differ on disease exposure and age in the same study design. Moreover, although there is some evidence to suggest that age dependent associations between gait speed and neurocognitive function may reflect recruitment of higher order functional brain networks with loss of automatic motor control (Allali et al. 2007; Clark 2015), there is little work on the functional brain networks that moderate this association. In one of the few studies in this area Lo et al. (2017) reported a positive association between gait speed and intrinsic functional connectivity within the frontoparietal network (FPN) in older healthy adults with slower than normal gait speed and mild cognitive dysfunction (not attributable to major neurological, musculoskeletal, or other medical conditions) (Lo et al. 2017). This is consistent with the idea that the connectivity of the FPN may play a crucial role in explaining reports of associations between executive function and gait speed in older adults (Coppin et al. 2006; Holtzer et al. 2006).

In this study we employed resting-state functional magnetic resonance imaging (rs-fMRI) to help uncover the neural link between slower gait and impaired cognitive function in PLWH. Resting state fMRI detects co-occurring fluctuations in blood-oxygen-level-dependent (BOLD) signals, reflecting changes in blood flow and oxygenation, indirectly linked to neuronal activity through neurovascular coupling (Logothetis et al. 2001). As rs-fMRI captures spontaneous, task-unrelated BOLD signal fluctuations, it provides fundamental insight via derived resting-state networks (RSNs) into the intrinsic organization and architecture of the brain (Biswal et al. 1995; Yeo et al. 2011).

The primary aim of this study is to test the hypothesis that gait speed and cognitive test performance will be robustly linked in a sample of PLWH (*N* = 17) in a low socio-economic status (SES) context, relative to seronegative controls (*N* = 22). To determine the degree to which the association between gait speed and neurocognitive function in PLWH is akin to what is seen in healthy older adults we also analysed data in the public domain from a large US-based cohort of older adults. Finally, we set out to conduct a preliminary analysis to determine whether HIV status moderated the relationship between intrinsic functional brain connectivity and both gait speed and cognitive test performance. Based on the literature reviewed above, we hypothesise that both slower gait and poorer executive function will be associated with weaker connectivity within the FPN, and between the FPN and the rest of the brain, with these effects particularly pronounced in PLWH.

## Methods

### Clinical trial number

Not applicable.

### Sample

This study is a secondary analysis of data from a study of the combined effect of HIV and binge drinking on neurocognitive function and the brain conducted at the University of Cape Town (Ipser et al. 2022). Participants were recruited from a community health centre in Khayelitsha, Cape Town, South Africa. PLWH who were ART-naïve or who had been on ART for less than one month were recruited at the clinic visit in which they were provided feedback on their HIV test results. HIV negative participants were recruited from the clinic’s voluntary HIV counselling and testing unit. Potentially eligible subjects were invited for additional clinical screening and cognitive testing in the Department of Psychiatry at Groote Schuur Hospital, Cape Town. Those who met further MRI-specific inclusion criteria (see below) were subsequently invited within two weeks of the first visit to undergo a MRI scan; at the Cape Universities Body Imaging Centre (CUBIC) at Groote Schuur Hospital.

All participants were between the ages 18 and 65 (inclusive) and had completed at least 7 years of formal education. Exclusion criteria included CNS disease, a history of head injury with loss of consciousness exceeding 30 min, treatment with psychotropic medication, a history of severe mental illness, active major depression or moderate to severe scores on the Beck Depression Inventory II (≥ 19), and a recent (12 month) substance abuse history for substances other than alcohol (assessed using the MINI, version 6 (Sheehan et al. 1998). Participants with contraindications for MRI, including claustrophobia and the presence of ferro-magnetic craniofacial implants, were excluded from the imaging visit.

For the purposes of this study, data was restricted to participants with a history of no or minimal drinking (≤ 2 drinks/day on at most 2 to 3 days per week over the past year) and for whom gait data were available (17 HIV+, 22 HIV-). One participant from the HIV- group had missing data for the motor domain and the derived global neurocognitive score. This participant was excluded from analyses involving these outcomes, but was included in models for the other neurocognitive domains. Rs-fMRI analyses were restricted to a subgroup of the participants who had scan data that passed quality control (11 HIV+, 11 HIV-). All participants provided informed consent. The study was conducted according to the principles of the Declaration of Helsinki (World Medical Association, 2013) and was approved by the UCT Human Research Ethics Committee (Ipser et al. 2022).

To assess the convergence of outcomes associated with HIV status and normal aging processes, we conducted an additional secondary analysis using publicly available data from the National Social Life, Health, and Aging Project (NSHAP). This replication analysis focused on the relationship between gait speed and neurocognitive performance in a large, nationally representative sample of older adults in the United States (*N* = 3196). Full details of this analysis, including sample characteristics and measures used, are provided in the supplementary material (see Section B).

### Clinical assessment

PLWH were required to have two positive results using independent rapid HIV test kits, with the ELISA test employed to reconcile discrepant test results. Furthermore, to avoid possible confounding effects of ART on brain health, PLWH participants eligible for inclusion were ART-naïve or had received ART for a period of no longer than 1 month, at testing. HIV viral load and CD4 count were assayed from blood samples. The lowest values when comparing these assays with those recorded from previous clinic visits, as provided by the National Health Laboratory Services, were recorded as nadir CD4 counts.

### Neurocognitive testing

This study utilized a Xhosa translation of a comprehensive cognitive battery that included tests for 7 different cognitive domains (Joska et al. 2012). These domain [and the tests used to assess each] were (1) speed of processing [Colour Trails 1 and Symbol Search (of the Wechsler Adult Intelligence Scale Fourth Edition, WAIS-III)], (2) language [Category Fluency (animals, fruits and vegetables)], (3) executive function [Colour Trails 2 and a modified version of the Stroop Colour and Word Test], (4) delayed recall [the Hopkins Verbal Learning Test and the Brief Visuospatial Memory Test], (5) learning [immediate recall scores from the two tests for delayed recall], (6) working memory [backward and forward versions of the Digit Span task of the WAIS-III] and (7), motor function on dominant and non-dominant hands [Grooved Pegboard].

### Assessment of gait

Neuro-medical examinations, including a timed gait test, were completed for each participant in the; study. Participants were timed, using a stopwatch, walking 10 yards (9.14 m) along a passage-way and then turning around and returning. The total time taken to walk 20 yards was used as an indicator of gait speed. The 10-yard distance was clearly marked, and participants were asked to walk at a good/fast pace and instructed not to run or ‘speed walk’. The task was repeated three times, and the average time taken across the three trials was used as the gait timing score for each participant.

### MRI acquisition

A 3 T Siemens Skyra MRI machine with a 32-channel head-coil was used to acquire structural T1-weighted MRI images, by applying a multi-echo magnetization-prepared rapid gradient echo (MPRAGE) sequence (TR = 2000 ms, TE = 1.69 ms, 3.55 ms, 5.41 ms, 7.27 ms, flip angle = 7°, FoV = 256 × 256 mm, voxel size: 1.0 × 1.0 × 1.5 mm). Whole brain rs-fMRI scan data was also acquired (TR/TE=2000ms/30ms; flip angle = 90 degrees; voxel size = 3.4 × 3.4 × 4mm3; 64 × 64 matrix; 33 slices, 308 volumes, 10 min). For the rs-fMRI sequence, participants were instructed to keep their eyes open whilst fixating on a cross displayed on a monitor in the scanner bore.

### Rs-fMRI pre-processing and quality control

This project employed the open-source Harmonized AnaLysis of Functional MRI pipeline (HALFpipe; Waller et al. 2022). A containerized version (v 1.2) of the HALFpipe pipeline was run on the UCT high performance computing (HPC) cluster and was applied to T1 weighted MPRAGE and resting state fMRI datasets.

HALFpipe’s pre-processing steps include slice timing correction, spatial smoothing of 6 mm, grand mean scaling of 10,000, and frequency-based temporal filtering (bandpass filtering using default filter cut-offs of 0.01 and 0.1 Hz). The influence of motion artifacts on the BOLD dataset was minimised through application of the ICA-AROMA (Pruim et al. 2015) and aCompCor (Behzadi et al. 2007) algorithms, in addition to regression of 6 motion parameters. The aCompCor algorithm performs principal component analysis to extract the 5 most prominent components from signal in white matter and cerebrospinal fluid, which are subsequently regressed from the BOLD signal.

Images were manually and independently checked for abnormalities by two raters (SS & JI), using the quality control interface provided by HALFpipe. Two subjects were excluded due to poor quality of scan data. An additional participant was retained after truncating their rs-fMRI timeseries at 8 min, to exclude motion-contaminated volumes towards the end of the scan sequence.

### Rs-fMRI seed selection and processing

HALFpipe was employed to generate whole-brain connectivity maps as well as pairwise correlation matrices using seeds placed in MNI (ICBM 2009c Nonlinear Asymmetric) space. Whole-brain connectivity and between network analyses were based on seeds corresponding to the 7 canonical RSN masks published in Yeo et al. 2011. These included seeds for the frontoparietal network (FPN), limbic network (LIM), visual network (VIS), default mode network (DMN), ventral attention network (VAN), dorsal attention network (DAN), and the somatomotor network (SMN). Within HALFpipe network connectivity maps were generated using FSL’s GLM tool by extracting the average BOLD time-series across voxels within a network seed and using this as a regressor for each individual voxel time series across the entire brain. This produces a Z score for each voxel, representing its connectivity strength with the network seed (Waller et al. 2022). Notably, data from 17 of the 22 participants in the local study failed to meet the minimal 80% overlap criterion set in HALFpipe across all nodes within the LIM (see below for description of derivation of nodes), likely due to BOLD signal attenuation known to affect inferior regions of the brain when using standard acquisition protocols. Accordingly, the limbic network was excluded from all further analyses.

The selected nodes for within-network connectivity were derived from the Power functional brain parcellation (Power et al. 2011). HALFpipe’s ‘atlas-based connectivity matrix’ feature was run to produce a correlation matrix of all pairwise combinations of all nodes from the atlas. Within the AFNI neuroimaging software suite (https://afni.nimh.nih.gov/), the Yeo et al. network masks were overlaid on the 264 nodes contained within the Power et al. (2011) atlas, to identify network-specific nodes. The network masks were eroded by two voxels prior to this step, to minimise the probability of the same nodes overlapping with multiple networks.

### Statistical analysis

#### Neurocognitive data

Neurocognitive domain scores were calculated by averaging Z scores for domain-specific tests, with the direction of scores from timed tasks reversed to ensure that higher scores for all tasks/domains reflect better performance. Scores for the motor domain were transformed using the exponential function, in order to better satisfy the assumption of normality required for parametric tests. The Z scores for individual domains were summed to produce a global NP score. Pearson’s correlation and t-tests were conducted to characterise strength of associations within the entire sample between the outcome variables (gait time, neurocognitive scores) and HIV status, as well as demographic variables (age and sex). The association between average gait time and neurocognitive domain Z scores within the PLWH and HIV seronegative groups was tested using non-parametric methods (Spearman’s correlation) due to the small number of participants in these analyses. Bonferroni correction was employed to adjust for multiple comparisons for analyses involving neurocognitive domains, with the corrected alpha set to 0.0071, computed as an unadjusted alpha of 0.05 divided by the number of domains tested (*N* = 7). Linear regression models were employed for the global neurocognitive score, as well as for each standardised domain score, to evaluate main effects of age, sex, gait time and HIV status, with follow-up models including an additional term to test for an interaction between HIV status and gait time on these outcomes.

#### fMRI data

The AFNI FATCAT toolbox was used to test the a priori hypothesis of group differences in the association of within-network FPN connectivity with gait time and neurocognitive function (domain and global scores) (Taylor 2013). Group differences for the other networks were assessed as exploratory analyses. Within-network correlation matrices were restricted to the top 15 most strongly inter-connected nodes for each network, to reduce computational burden. The correlation matrices were input to AFNI’s 3dMVM tool, via the FATCAT toolbox, to separately determine group differences in the association of connectivity between nodes within each network and gait time and neurocognitive scores. This multivariate regression modelling approach allows for correlations between each pairwise combination of nodes to be treated as within-subject repeated-measure factors, while simultaneously adjusting for the effect of continuous covariates (Chen et al. 2014). Convergence in node-pairs that demonstrated statistically significant group (PLWH, HC) interaction effects in both the gait speed and neurocognitive models were used to provide a more spatially localised indication of the neural substrate of HIV-specific differences in gait-speed-associated neurocognitive-function.

The impact of HIV status on the association of gait speed with between – network connectivity was also assessed, as part of an exploratory analysis. Connectivity estimates passed to FATCAT for this purpose took the form of a correlation matrix of associations between the time-series for each network, computed as the average of the time-series of constituent network nodes. In recognition of the small sample with rs-fMRI data, no additional covariates, including sex and age, were included in any of the FATCAT models run, in an attempt to bolster power to detect interaction effects of interest.

## Results

### Sample characteristics

A total of 39 participants (17 PLWH and 22 HIV seronegative controls) were included in the neurocognitive and gait data analyses. No significant differences in age, sex, years of education, gait time or global neurocognitive score were observed between groups (*p* > 0.05). Demographic data of all the subjects, as well as clinical variables for the PLWH group are shown in Table 1. All participants identified as Black African. Older subjects took significantly longer to complete the gait task (*R* = 0.317, *p* = 0.049), and also performed worse on average for the global neurocognitive score and for specific neurocognitive domains (speed of processing, memory, learning, motor function; see Table S1). Male subjects demonstrated better performance in the memory domain than females (see Table S1), with higher nadir CD4 count associated with better motor function (see Table S2). No statistically significant associations were observed between any of the other demographic/clinical variables and either gait time or neurocognitive domain scores.

**Table 1:** Sample characteristics

	PLWH (*N* = 17)	HIV seronegative (*N* = 22)	Test	*p*-value
Age	39.06 (9.94)	38.95 (8.43)	Z = 0.241	0.809
Sex: Female	9 (52.94%)	15 (68.18%)	X^2^ = 0.407	0.523
Years of education	10.47 (1.42)	10.59 (1.65)	Z = 0.277	0.782
Currently taking ART	6 (35.29%)	–	–	–
Detectable HIV viral load	8 (47.06%)	–	–	–
HIV viral load (log transformed)	8.14 (1.91)	–	–	–
Nadir CD4 count	439.8 (174.19)	–	–	–
Gait time (seconds)	10.13 (1.18)	9.83 (1.37)	Z = -0.510	0.619
Global Neurocognitive score (Z)	0.09 (0.56)	0.20 (0.51)	Z = 0.866	0.399

For continuous variables (age, years of education, HIV-load, nadir CD4, gait time), results are represented as: mean (standard deviation). For categorical variables, results are presented as sample size (percentage). The Mann-Whitney was used to test for differences between groups in age, years of education, gait time and global neurocognitive score. Sex differences were assessed with the Chi-squared test. Note: ART use and detectability of viral load data missing for 8 participants, HIV viral load (log transformed) data missing for 2 participants, nadir CD4 count data missing for 1 participant. NP, neuropsychological; HIV, human immunodeficiency virus; HIV+, HIV seropositive group; HIV-, seronegative control group; ART, antiretroviral therapy

### Associations between gait speed and global neurocognitive function

Longer gait time predicted worse global neurocognitive function across the entire sample, after adjusting for age and sex, with each additional second taken to complete the gait task, on average, associated with a reduction by 0.161 standard deviations on the global neurocognitive Z score (stand beta = -0.393, *p* = 0.012; see Table 2). As evident in Table 1, HIV status was not associated with either global neurocognitive function or gait time.

**Fig. 1:**
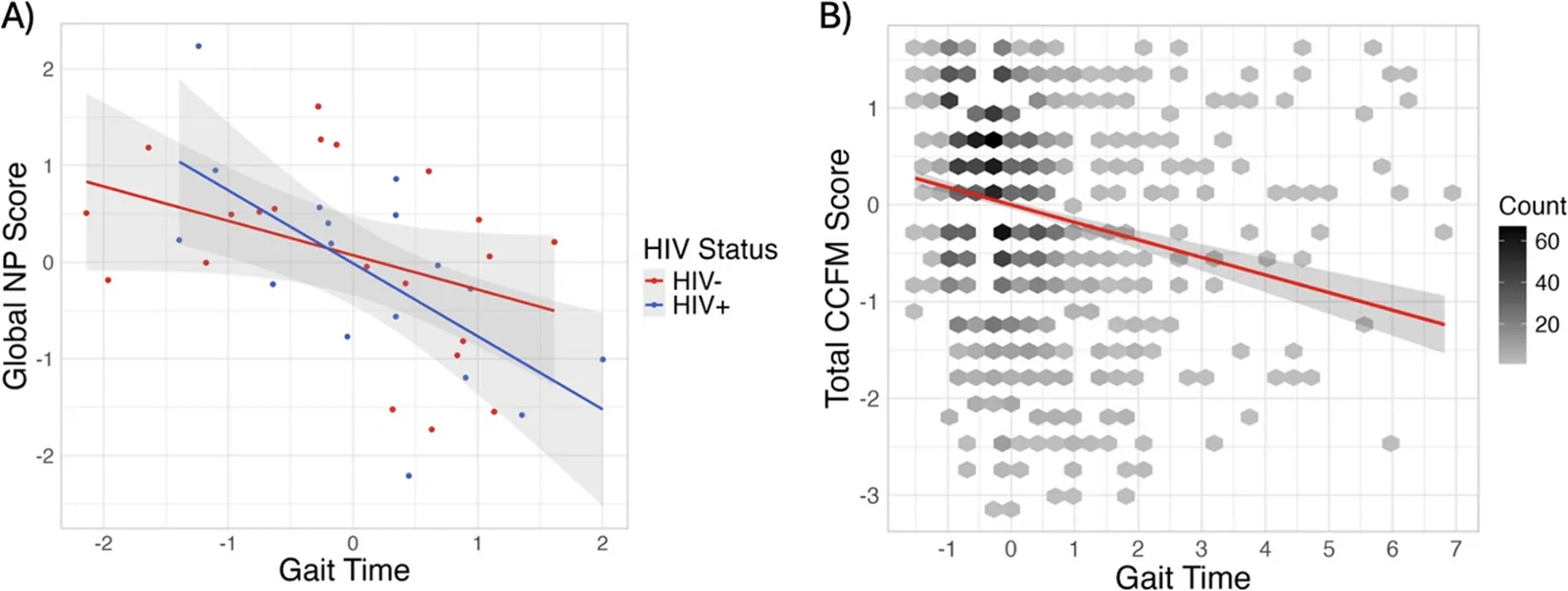
(**A**) Scatter plot illustrating relationship between gait time and global neurocognitive scores in the LMIC dataset, separated by HIV status. Both gait time and neurocognitive performance scores are presented as standardized Z-scores on both axes. Scatter Regression lines are shown with shaded areas indicating 95% confidence intervals. *One participant from the HIV- group was excluded due to having missing data for the motor function domain and therefore for the Global neurocognitive score (*N* = 38). (**B**) Hexagon plot illustrating relationship between gait time and total CCFM scores in the NSHAP dataset (*N* = 1987), graded by frequency (count). Regression line shown with shaded area indicating 95% confidence intervals

The observation that, after adjusting for individual differences in sex and age, gait time was a significant predictor of global neurocognitive function was replicated in the NSHAP dataset, for which a statistically significant negative association was observed between time to complete the gait task and the total scores on the CCFM, an adaption of the MoCA neurocognitive screen (stand. beta = -0.105, *p* < 0.001; see Table 2). Model statistics and a visualisation of the relationship between global cognitive function and gait time can be found for both the LMIC and public domain datasets in Table 2 and in Fig. 1), respectively.

**Table 2:** Linear models for independent effects of age, sex and gait time as predictors of global neurocognitive scores for the HIV Study cohort (*N* = 38) and for total CCFM scores in the weighted NSHAP Wave II sample (*N* = 2081.39)

	Linear Model Results						
	b	SE for b	β	SE for β	t-value	*p*-value	Adj. *r*^2^
All participants (*N* = 38)							0.31
Gait time	-0.161	0.06	-0.393	0.15	-2.66	**0.012**	
Age	-0.020	0.01	-0.345	0.14	-2.38	**0.023**	
Sex (male)	-0.016	0.15	-0.030	0.28	-0.10	0.918	
HIV+ subgroup (*N* = 17)							0.47
Gait time	-0.284	0.09	-0.600	0.19	-3.12	**0.008**	
Age	-0.015	0.01	-0.267	0.19	-1.40	0.186	
Sex (male)	0.313	0.20	0.560	0.36	1.57	0.140	
HIV- subgroup (*n* = 21)							0.28
Gait time	-0.125	0.08	-0.339	0.22	-1.51	0.149	
Age	-0.029	0.01	-0.483	0.21	-2.32	**0.033**	
Sex (male)	-0.280	0.23	-0.553	0.46	-1.21	0.245	
NSHAP cohort (N = *2081.39*)							0.13
Gait time	-0.206	0.04	-0.105	0.02	-4.80	**< 0.001**	
Age	-0.112	0.01	-0.239	0.02	-11.47	**< 0.001**	
Sex (female)	0.671	0.13	0.211	0.04	5.16	**< 0.001**	

Linear model includes the independent effects of gait time, age and sex as predictor variables and the cognitive domain score as the outcome variable. CCFM: Chicago Cognitive Function Measure; b: coefficient estimate; β: standardized coefficient; SE: standard error; Adj: adjusted. Bold indicates p < 0.05, unadjusted

### Identifying neurocognitive domains sensitive to gait speed and HIV status

Table 3 contains the results of Spearman’s correlation tests between gait time and neurocognitive domain Z scores, presented for the entire sample as well as separately for HIV seronegative and seropositive participants. All test coefficients were negative, indicating that longer time to complete the timed gait task was associated with poorer neurocognitive function. Statistically significant correlations were observed between gait time and language (Rho = -0.407, *p* = 0.010), memory (Rho = -0.531, *p* < 0.001), learning (Rho = -0.375, *p* = 0.019) and motor function (Rho = -0.420, *p* = 0.009) across the entire sample. Of these findings, only the association between memory function and gait speed remained after Bonferroni correction for multiple comparisons. When stratified by group, statistically significant associations were detected for the memory domain for both groups, and for the motor domain in PLWH participants only.

**Table 3:** Spearman’s correlations between gait time and cognitive domain scores for UCT HIV study dataset (*n* = 39)

Cognitive Domain	Correlation With Gait Time					
	**Whole sample** (*n* = 39)		**HIV+ group** (*n* = 17)		**HIV- group** (*n* = 22)	
	Rho	*p*-value	Rho	*p*-value	Rho	*p*-value
Speed of processing	-0.283	0.081	-0.275	0.286	-0.259	0.243
Language	**-0.407**	**0.010**	-0.319	0.211	-0.405	0.063
Executive function	-0.277	0.088	-0.401	0.111	-0.158	0.482
Memory	**-0.531**	**< 0.001**	**-0.580**	**0.015**	**-0.449**	**0.036**
Working memory	-0.268	0.099	-0.382	0.131	-0.145	0.519
Learning	**-0.375**	**0.019**	-0.405	0.106	-0.353	0.107
Motor function	**-0.420**	**0.009**	**-0.586**	**0.013**	-0.326	0.149

NP; neuropsychological. p-values are not corrected for multiple comparisons. Bold indicates p < 0.05, unadjusted

Table 4 summarises the results from linear models designed to test the main effects of gait time and HIV status for global neurocognitive function as well as each of the neurocognitive domain scores while accounting for individual differences in age and sex (see Table S3 for effect estimates for all model terms). A main effect was observed for gait time, with slower gait associated with poorer performance across the entire sample for the language and memory domain scores (beta = -0.221, *p* = 0.047, and beta = -0.355, *p* = 0.006, respectively). Again, only the memory domain effect remained significant after Bonferroni adjustment for multiple comparisons. There was no evidence that the association between gait time and global neurocognitive or domain score varied as a function of HIV status (Table S3), although there was sub-threshold evidence that the association between gait speed and memory function may differ between PLWH and seronegative participants (beta = -0.405, *p* = 0.10).

**Table 4:** Linear models for effect of gait time and HIV status on cognitive domain scores, after adjusting for age and sex (*n* = 39)

Predictor variables for each cognitive domain	β	SE	t-value	*p*-value	Adj. *r*^2^
*Global NP score*					0.291
HIV status (+)	-0.116	0.280	-0.413	0.683	
Gait time	-0.385	0.151	-2.550	**0.016**	
*Speed of processing*					0.084
HIV status (+)	-0.042	0.249	-0.170	0.866	
Gait time	-0.130	0.131	-0.985	0.332	
*Language*					0.177
HIV status (+)	-0.345	0.202	-1.708	0.097	
Gait time	-0.221	0.107	-2.066	**0.047**	
*Executive function*					0.036
HIV status (+)	-0.370	0.284	-1.303	0.201	
Gait time	-0.100	0.150	-0.667	0.509	
*Memory*					0.318
HIV status (+)	0.083	0.231	0.358	0.723	
Gait time	-0.355	0.122	-2.908	**0.006**	
*Working memory*					0.095
HIV status (+)	0.211	0.225	0.939	0.354	
Gait time	-0.192	0.119	-1.616	0.115	
*Learning*					0.106
HIV status (+)	-0.137	0.243	-0.564	0.577	
Gait time	-0.165	0.128	-1.285	0.208	
*Motor function*					0.221
HIV status (+)	0.205	0.208	0.988	0.330	
Gait time	-0.218	0.112	-1.950	0.060	

Trans, Fisher Z transformed values were used for the Motor function domain; β; standardized coefficient estimate; SE: standard error; NP: neuropsychology; Bold indicates p < 0.05, unadjusted

### Intrinsic functional brain connectivity

#### Sample characteristics of the neuroimaging dataset

The 11 PLWH participants and 11 seronegative controls included in the rs-fMRI analyses were comparable in terms of age, sex, and years of education. Moreover, participants included in the neuroimaging analysis did not differ substantially on demographic or clinical variables from the larger sample with neurocognitive data (see Table S5).

#### Within-network connectivity

A statistically significant interaction effect between PLWH group and gait time was observed for the omnibus model testing connectivity between all 15 FPN nodes (X^2^ = 9.888, *p* = 0.002). No HIV status - gait interactions were identified in exploratory within-network analyses for any of the remaining networks. In subsequent within-network models of FPN connectivity that (separately) substituted global neurocognitive and domain scores for gait time, executive function was the only domain for which an interaction on FPN connectivity with HIV status was observed (X^2^ = 10.303; *p* = 0.001). Given the observation that memory function was the most robust predicter of gait time for this cohort, it is noteworthy that a trend interaction effect was also observed for HIV status and memory performance on connectivity within the FPN (X^2^ = 3.386, *p* = 0.066).

**Fig. 2:**
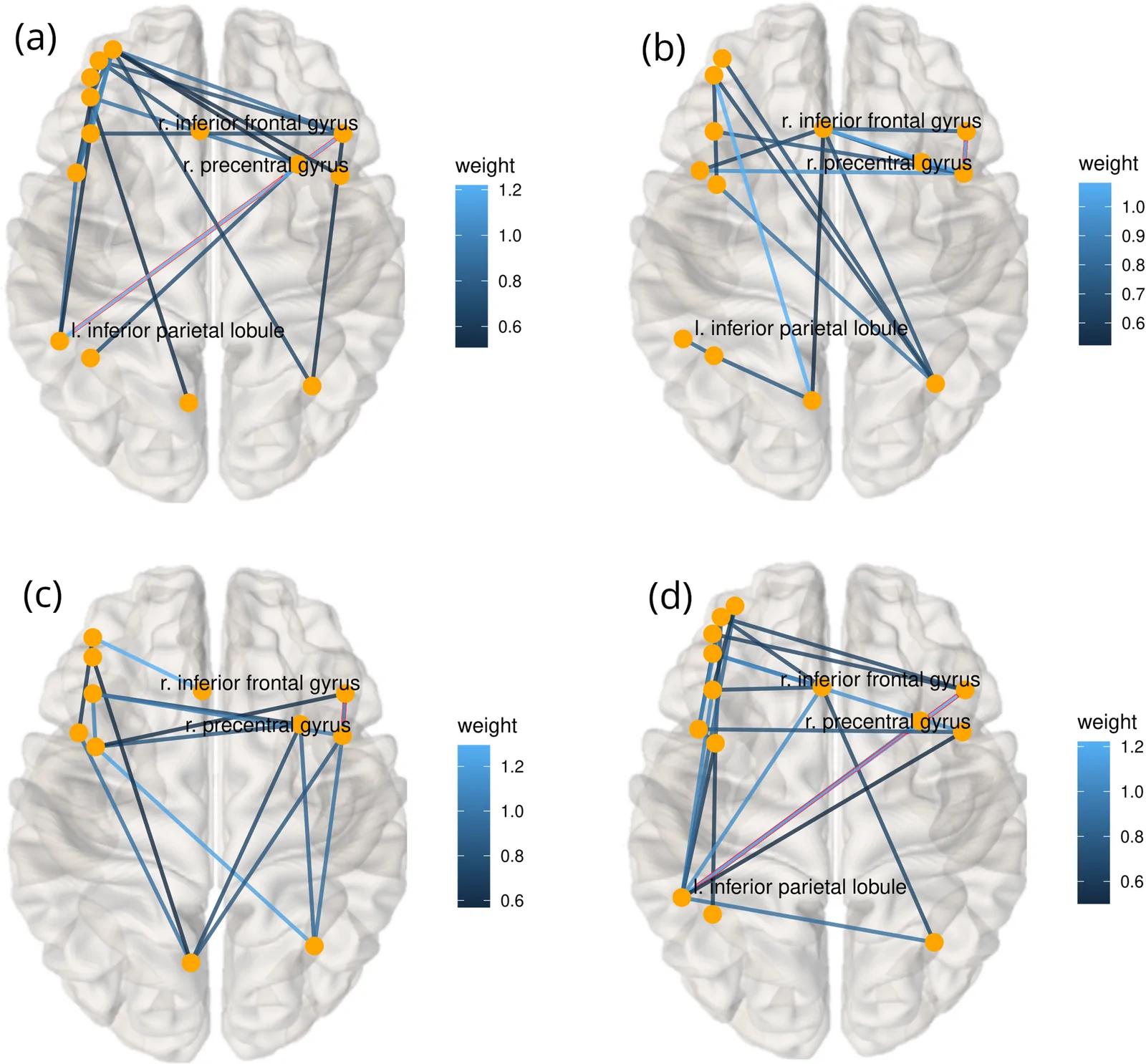
Brain plots illustrating group differences in associations of FPN within-network connectivity with gait time and executive function. All differences are thresholded at a minimum value of 0.5, corresponding to a “large” effect size according to the Cohen (1988) conventions. Group differences in associations between gait time and FPN connectivity that are (**a**) larger and (**b**) smaller in PLWH than HIV seronegative participants. Group differences in associations between executive function scores and FPN connectivity that are (**c**) larger and (**d**) smaller in PLWH than HIV seronegative participants. Lines denoting connections where group differences are observed for both gait and executive function are highlighted in red

The convergence analysis identified 2 FPN node-pairs where connectivity was differentially associated with both gait time *and* executive function in PLWH participants compared to seronegative controls (see Fig. 2). Both pairs involving a node located in the right inferior frontal gyrus (r. IFG) (Table 5). Specifically, for the HIV+ group only, both slower gait speed and lower executive function scores were associated with less connectivity between the r. IFG and the right precentral gyrus (Rho = -0.645; *p* = 0.037 and Rho = 0.691; *p* = 0.023, respectively) and with greater connectivity between the r. IFG and the left inferior parietal lobe (Rho = 0.809, *p* = 0.004 and Rho = -0.818; *p* = 0.004, respectively).

**Table 5:** Group differences in the association of connectivity of the FPN node pairs identified from the convergence analysis with gait time and executive function

Node-pair within FPN	Correlation between gait time and pair-wise connectivity				Correlation between executive function and pair-wise connectivity			
	PLWH		Controls		PLWH		Controls	
	Rho	*p*-value	Rho	*p*-value	Rho	*p*-value	Rho	*p*-value
r. IFG - lIPL	0.809	**0.004**	-0.309	0.356	-0.818	**0.004**	0.573	0.071
r. IFG - r. PG	-0.645	**0.037**	0.455	0.163	0.691	**0.023**	-0.427	0.193

*FPN*: frontoparietal network; *l. IPL*: left inferior parietal lobule; *r. IFG*: right inferior frontal gyrus; *r. PG*: right precentral gyrus

Bold indicates p < 0.05, unadjusted

#### Between-network connectivity

There was no evidence of a main effect of gait speed on connectivity between the 6 networks (X^2^ = 0.884, *p* = 0.347), or that this association was impacted by HIV status (X^2^ = 0.842, *p* = 0.359).

## Discussion

In this study we set out to assess whether HIV status moderates the extent to which gait speed indexes cognitive performance in adults, an important first step in understanding whether gait speed could have clinical utility in screening for HABI in PLWH. We compared the patterns observed with those derived from a large public domain dataset of healthy older adults, to interrogate the extent to which HIV-associated accentuated ageing might inform our findings. As a further step towards gaining mechanistic insight into the relationship between gait speed and cognitive function, we tested the hypothesis that this association would be moderated by intrinsic connectivity of the FPN. As predicted, a stronger relationship was observed in PLWH than seronegative controls between gait speed and neurocognitive function (Table 2), though this difference did not achieve statistical significance when tested directly (see Table S3). Moreover, we observed evidence to support our hypothesis that the relationship between gait time and intrinsic functional brain connectivity differs as a function of HIV serostatus. This finding was specific to the FPN and was not observed for the remaining five RSNs assessed in exploratory analyses.

The observation that executive function was the only domain for which performance differentially predicted connectivity of the FPN in PLWH compared to HIV seronegative controls is consistent with the FPN’s involvement as a possible mediator of the relationship between gait speed and executive function. This finding also aligns with the observation in a recent study that age-related decline in executive function was linked to frontoparietal volume (Yao et al. 2020), as well as the findings from other studies that executive function mediates the stability and velocity of gait in older adults through maintaining attention to walking and inhibiting distracting stimuli (Coppin et al. 2006; Holtzer et al. 2006). Indeed, given the literature indicating the involvement of the FPN in externally-oriented attentional processes (Markett et al. 2014), individual differences in intra-network connectivity of the FPN may also underly findings from studies employing cognitive-motor dual-task paradigms, including one in a South African PLWH cohort (Berner et al. 2021), in which the performance of attention-demanding tasks is associated with reductions in gait speed with or without decrements in cognitive performance (Belghali et al. 2017).

Connectivity for two seed-pairs within the FPN, both involving the r. IFG, were significantly and exclusively correlated with both gait speed and executive function in PLWH in this study. These findings seem to suggest that, in PLWH, both slower walking speed (i.e. more time taken to complete the gait task), as well as poorer executive function, predict greater connectivity between frontoparietal regions and less frontal cortical connectivity to the precentral gyrus. Conversely, successful performance on these tasks in PLWH may reflect disengagement of the frontoparietal network in favour of greater reliance on motor-specific brain regions to successfully execute both walking and executive function tasks. Accordingly, one possible explanation for the pattern of findings observed in this study is that compromised frontoparietal brain function in PLWH puts patients who are not able to leverage compensatory brain networks at a relative disadvantage in terms of executive function.

Altered between-network functional connectivity between the FPN and SMN has previously been reported in older adults with mild NCI and slower gait speed (Hsu et al. 2019). Although we were not able to replicate this finding when we assessed connectivity between networks, it should be noted that one of the nodes whose connectivity to the r. IFG predicted both gait time and executive function, i.e., the precentral gyrus, is typically considered to be a key region of the motor cortex. In this study, we used masks derived from the Yeo 2011 functional parcellation to define networks, which incorporates the precentral gyrus as part of the FPN. This apparent discrepancy could be explained by the previous observation that the FPN and SMN overlap to some extent anatomically (Hsu et al. 2014) and functionally, which may account for the involvement of both networks in top-down control of motor planning and motor execution (Wise et al. 1997; Wymbs et al. 2012). Hsu et al. suggest that decreased connectivity between these two networks during resting state reflects less ability to harness motor preparatory inputs from FPN to the SMN, in anticipation of motor performance. It is also pertinent to note that performance of the timed gait task, and the Colour Trails task, one of the tests included in the executive function domain in this study, both rely on the preservation of motor *and* executive function abilities. Combined, these findings suggest that the r. IFG may be a candidate neural substrate underlying compensatory mechanisms that have previously been implicated in dual task paradigms as supporting executive function and walking speed in PLWH within a low-SES context (Berner et al. 2021).

This study’s findings provided little support for the hypothesis that the relationship between gait speed and performance was dependent on HIV status for global cognitive function, or for any of the neurocognitive domains, after accounting for individual differences in age and sex. Nevertheless, results from bivariate analyses reported in this paper suggest that a stronger negative correlation exists between memory function and gait time in PLWH compared to seronegative controls (Table 3). This is consistent with the preliminary finding of a subthreshold interaction effect for HIV status and gait speed on memory function (beta = -0.405, *p* = 0.100; see Table S3). These findings need replication in larger well-powered studies, potentially justifying the use of the timed gait task in deciding whether to conduct further screening for memory impairments in PLWH in a clinical setting.

In summary, the findings reported in this paper, both with respect to the association of gait timing with neurocognitive function and with associated brain substrates, are broadly consistent with those reported in the literature. This is particularly striking given that the sample contributing rs-fMRI data in this study was considerably younger, on average, than the older adult samples typically assessed in studies linking gait to neurocognitive function. This point is further underscored by the general pattern of convergence of findings from the local dataset and the NSHAP cohort (see Table S8 & S9), despite the fact that the latter sample were considerably older on average than the local low-SES sample (mean age = 71.9 versus 39 years, respectively). Indeed, gait disturbances were observed in the only published report of gait in PLWH in South Africa in a comparable age group (mean age = 33.8 years). These findings support accentuated ageing in PLWH as a possible mechanism linking abnormal FPN connectivity with poorer executive function and slower gait speed in this clinical population.

### Study limitations and future directions

The consistency of our findings with those previously reported in the scientific literature provides some confidence in their validity. This is further reinforced by our ability to demonstrate robustness of the association between gait speed and global neurocognitive function in both LMIC and high income country settings. Nevertheless, there are a number of limitations to this study design that should be taken into account when interpreting the results presented in this paper. This includes the small sample size of the LMIC dataset, particularly for the rs-fMRI analysis, which limited the power of our models to detect HIV-gait interactions. The small sample also prevented us from including clinical variables of interest, such as HIV viral load or CD4 count, in regression models. Nevertheless, inspection of our data suggest that these variables had little influence on the association of neurocognitive function with gait time. Although the confounding effects of ART exposure on brain health were not considered in this study, this was partially mitigated by only including participants who were ART-naïve or had been on ART for no longer than a month at the time of testing. It should also be emphasised that, while the LMIC sample conformed to stringent selection criteria, evidence for an exclusive association between gait, neurocognitive function, and RSN connectivity in PLWH in our study cannot be solely attributed to the direct manifestations of HIV infection (i.e. HABI), as other factors not considered in this study may contribute to cognitive decline in this patient population. Finally, consideration of the NSHAP dataset as a replication cohort is somewhat undermined by differences in the cognitive instruments employed by the respective studies, as the CCFM employed in the NSHAP cohort is based on the MoCA, and is more accurately considered a tool to screen for neurocognitive impairment than a full neurocognitive battery, as administered in the LMIC parent study. Nevertheless, studies of healthy older adults that have employed more comprehensive test batteries have also identified executive function and memory, as well as speed of processing, as the domains most strongly associated with gait speed (Watson et al. 2010; Gale et al. 2014).

## Conclusion

In conclusion, this study’s findings provide insight into the complex interplay between gait speed, neurocognitive function, and resting-state brain connectivity in PLWH. We are able to replicate, for the first time, the observation of a significant association between gait time and global neurocognitive function in two independent datasets, spanning substantially different socioeconomic and cultural settings. These associations were larger in magnitude in a cohort recruited from a LMIC setting than those recruited from a high income country setting, and, as hypothesised, were particularly apparent in PLWH. The possibility that accelerated ageing in PLWH may be one possible mechanism through which HIV-specific associations between gait and neurocognitive function manifest should be investigated in future studies. Our results further indicate that the integrity of the FPN, and the right IFG in particular, may mediate this association. In summary, our findings support the potential role of gait speed as an early indicator of neurocognitive changes in PLWH. Further exploration into the neural underpinnings of gait-cognition interactions in this patient population is warranted.

## Supplementary Information

Below is the link to the electronic supplementary material.

Supplementary Material 1 (DOCX 393 KB)

## Data Availability

Anonymised data from the LMIC cohort will only be made available to those parties with a data usage and transfer agreement approved by the UCT Human Research Ethics Committee. The public domain data used for the secondary replication analysis are available at https://www.icpsr.umich.edu/web/NACDA/studies/34921 (DOI:10.3886/ICPSR34921.v5).
