# Supplementary material for: Evidence that intrinsic brain connectivity moderates the association between gait and neurocognitive function in people living with HIV
Source: J Neurovirol. 2026 Oct 13;32(6):46. doi: 10.1007/s13365-026-01348-0 (PMC13661632; doi:10.1007/s13365-026-01348-0)
Supplement: Supplementary file 1 [file 13365_2026_1348_MOESM1_ESM.docx]

**Supplementary material**

**Section A:**

**Table S1:** Bivariate analyses of associations between age, sex and HIV status, and the outcome variable (cognitive domains and gait time) in local dataset (an exponential function was applied to motor domain scores, to correct for non-normality)

|  | | Age | | Sex | | | HIV status | | |
| --- | --- | --- | --- | --- | --- | --- | --- | --- | --- |
|  |  | *r* | p-value | Mean for females | Mean for males | p-value | Mean for HIV- | Mean for HIV+ | p-value |
| Global neurocognitive function | | -0.479 | **0.002** | 0.105 | 0.229 | 0.417 | 0.203 | 0.094 | 0.537 |
|  | Speed of processing | -0.393 | **0.013** | -0.037 | 0.058 | 0.692 | 0.035 | -0.046 | 0.761 |
|  | Language | -0.281 | 0.083 | -0.174 | -0.072 | 0.632 | 0.037 | -0.357 | 0.068 |
|  | Executive function | -0.269 | 0.098 | -0.107 | 0.053 | 0.559 | 0.119 | -0.258 | 0.188 |
|  | Memory | -0.349 | **0.030** | -0.263 | 0.403 | **0.008** | -0.037 | 0.032 | 0.809 |
|  | Working memory | -0.229 | 0.160 | -0.020 | -0.333 | 0.182 | -0.181 | -0.088 | 0.694 |
|  | Learning | -0.336 | **0.037** | -0.143 | 0.222 | 0.178 | 0.057 | -0.080 | 0.572 |
|  | Motor | -0.413 | **0.010** | 1.482 | 1.271 | 0.319 | 1.356 | 1.452 | 0.691 |
| Gait time | | 0.317 | **0.049** | 10.148 | 9.657 | 0.284 | 9.825 | 10.132 | 0.458 |

Pearson’s correlation was conducted to test correlations with age. Effects of sex and HIV status were tested with t-tests. Bold indicates p < 0.05, unadjusted.

**Table S2:** Bivariate analyses of associations HIV-related clinical variables and the outcome variable (cognitive domains and gait time) in local dataset (an exponential function was applied to motor domain scores, to correct for non-normality)

|  | | HIV viral load  (log transformed) | | Nadir CD4 count | |
| --- | --- | --- | --- | --- | --- |
|  |  | *r* | p-value | *r* | p-value |
| Global neurocognitive function | | 0.086 | 0.763 | 0.138 | 0.609 |
|  | Speed of processing | 0.143 | 0.611 | -0.153 | 0.571 |
|  | Language | 0.234 | 0.401 | 0.187 | 0.488 |
|  | Executive function | -0.125 | 0.658 | -0.032 | 0.908 |
|  | Memory | 0.136 | 0.630 | 0.153 | 0.571 |
|  | Working memory | 0.161 | 0.566 | 0 | 1 |
|  | Learning | 0.050 | 0.859 | -0.044 | 0.870 |
|  | Motor | -0.054 | 0.853 | 0.541 | **0.033** |
| Gait time | | -0.025 | 0.930 | -0.127 | 0.640 |

Spearman’s correlation was conducted to test correlations of outcome variables with log transformed HIV viral load and nadir CD4 count. Bold indicates p < 0.05, unadjusted.

**Table S3:** Linear models for independent effects of age, sex, gait time and HIV status (Model 1) and interactive effects of HIV and gait time (Model 2) on cognitive domain scores (n = 39).

|  | | **Model 1** | | | | | **Model 2** | | | | | |
| --- | --- | --- | --- | --- | --- | --- | --- | --- | --- | --- | --- | --- |
|  |  | $\beta$ | SE | t-value | p-value | Adj. r^2^ | $\beta$ | SE | t-value | p-value | Adj. r^2^ |  |
| ***Global score*** | |  |  |  |  | 0.291 |  |  |  |  | 0.311 |  |
|  | Age | -0.345 | 0.147 | -2.354 | **0.025** |  | -0.337 | 0.145 | -2.333 | **0.026** |  |  |
|  | Sex (male) | -0.010 | 0.291 | -0.034 | 0.973 |  | 0.105 | 0.298 | 0.353 | 0.727 |  |  |
|  | HIV status (+) | -0.116 | 0.280 | -0.413 | 0.683 |  | -0.108 | 0.276 | -0.391 | 0.699 |  |  |
|  | Gait time | -0.385 | 0.151 | -2.550 | **0.016** |  | -0.222 | 0.189 | -1.173 | 0.249 |  |  |
|  | HIV*Gait |  |  |  |  |  | -0.415 | 0.296 | -1.405 | 0.170 |  |  |
| ***Speed of processing*** | |  |  |  |  | 0.084 |  |  |  |  | 0.065 |  |
|  | Age | -0.270 | 0.130 | -2.087 | **0.045** |  | -0.266 | 0.131 | -2.027 | 0.051 |  |  |
|  | Sex (male) | -0.029 | 0.258 | -0.113 | 0.910 |  | 0.009 | 0.270 | 0.032 | 0.975 |  |  |
|  | HIV status (+) | -0.042 | 0.249 | -0.170 | 0.866 |  | -0.042 | 0.252 | -0.166 | 0.870 |  |  |
|  | Gait time | -0.130 | 0.131 | -0.985 | 0.332 |  | -0.075 | 0.167 | -0.445 | 0.659 |  |  |
|  | HIV*Gait |  |  |  |  |  | -0.147 | 0.268 | -0.548 | 0.588 |  |  |
| ***Language*** | |  |  |  |  | 0.177 |  |  |  |  | 0.155 |  |
|  | Age | -0.116 | 0.105 | -1.100 | 0.279 |  | -0.113 | 0.107 | -1.062 | 0.296 |  |  |
|  | Sex (male) | 0.037 | 0.209 | 0.178 | 0.860 |  | 0.057 | 0.219 | 0.258 | 0.798 |  |  |
|  | HIV status (+) | -0.345 | 0.202 | -1.708 | 0.097 |  | -0.345 | 0.205 | -1.684 | 0.102 |  |  |
|  | Gait time | -0.221 | 0.107 | -2.066 | **0.047** |  | -0.193 | 0.136 | -1.416 | 0.166 |  |  |
|  | HIV*Gait |  |  |  |  |  | -0.075 | 0.218 | -0.345 | 0.733 |  |  |
| ***Executive function*** | |  |  |  |  | 0.036 |  |  |  |  | 0.051 |  |
|  | Age | -0.193 | 0.147 | -1.311 | 0.199 |  | -0.182 | 0.147 | -1.242 | 0.223 |  |  |
|  | Sex (male) | 0.123 | 0.294 | 0.418 | 0.679 |  | 0.218 | 0.301 | 0.724 | 0.474 |  |  |
|  | HIV status (+) | -0.370 | 0.284 | -1.303 | 0.201 |  | -0.368 | 0.282 | -1.306 | 0.201 |  |  |
|  | Gait time | -0.100 | 0.150 | -0.667 | 0.509 |  | 0.040 | 0.187 | 0.214 | 0.832 |  |  |
|  | HIV*Gait |  |  |  |  |  | -0.372 | 0.300 | -1.239 | 0.224 |  |  |
| ***Memory*** | |  |  |  |  | 0.318 |  |  |  |  | 0.353 |  |
|  | Age | -0.148 | 0.120 | -1.231 | 0.227 |  | -0.135 | 0.117 | -1.157 | 0.256 |  |  |
|  | Sex (male) | 0.472 | 0.239 | 1.980 | 0.056 |  | 0.577 | 0.240 | 2.399 | **0.022** |  |  |
|  | HIV status (+) | 0.083 | 0.231 | 0.358 | 0.723 |  | 0.085 | 0.225 | 0.377 | 0.709 |  |  |
|  | Gait time | -0.355 | 0.122 | -2.908 | **0.006** |  | -0.202 | 0.149 | -1.355 | 0.185 |  |  |
|  | HIV*Gait |  |  |  |  |  | -0.405 | 0.239 | -1.693 | 0.100 |  |  |
| ***Working memory*** | |  |  |  |  | 0.095 |  |  |  |  | 0.091 |  |
|  | Age | -0.137 | 0.117 | -1.175 | 0.248 |  | -0.131 | 0.117 | -1.112 | 0.274 |  |  |
|  | Sex (male) | -0.461 | 0.233 | -1.980 | 0.056 |  | -0.405 | 0.242 | -1.675 | 0.103 |  |  |
|  | HIV status (+) | 0.211 | 0.225 | 0.939 | 0.354 |  | 0.213 | 0.226 | 0.942 | 0.353 |  |  |
|  | Gait time | -0.192 | 0.119 | -1.616 | 0.115 |  | -0.109 | 0.150 | -0.730 | 0.470 |  |  |
|  | HIV*Gait |  |  |  |  |  | -0.220 | 0.240 | -0.916 | 0.366 |  |  |
| ***Learning*** | |  |  |  |  | 0.106 |  |  |  |  | 0.080 |  |
|  | Age | -0.188 | 0.126 | -1.494 | 0.144 |  | -0.186 | 0.128 | -1.456 | 0.155 |  |  |
|  | Sex (male) | 0.268 | 0.251 | 1.067 | 0.294 |  | 0.282 | 0.263 | 1.072 | 0.291 |  |  |
|  | HIV status (+) | -0.137 | 0.243 | -0.564 | 0.577 |  | -0.136 | 0.246 | -0.555 | 0.583 |  |  |
|  | Gait time | -0.165 | 0.128 | -1.285 | 0.208 |  | -0.143 | 0.163 | -0.878 | 0.386 |  |  |
|  | HIV*Gait |  |  |  |  |  | -0.057 | 0.262 | -0.216 | 0.830 |  |  |
| ***Motor function*** | |  |  |  |  | 0.221 |  |  |  |  | 0.234 |  |
|  | Age | -0.245 | 0.109 | -2.255 | **0.031** |  | -0.240 | 0.108 | -2.224 | **0.033** |  |  |
|  | Sex (male) | -0.403 | 0.216 | -1.868 | 0.071 |  | -0.326 | 0.222 | -1.469 | 0.152 |  |  |
|  | HIV status (+) | 0.205 | 0.208 | 0.988 | 0.330 |  | 0.210 | 0.206 | 1.022 | 0.315 |  |  |
|  | Gait time | -0.218 | 0.112 | -1.950 | 0.060 |  | -0.110 | 0.141 | -0.777 | 0.443 |  |  |
|  | HIV*Gait |  |  |  |  |  | -0.276 | 0.220 | -1.253 | 0.219 |  |  |

Trans, Fisher Z transformed values were used for the Motor function domain; b, coefficient estimate; SE, standard error; Adj., adjusted. Bold indicates p < 0.05, unadjusted. * indicates an interactive term.

**Table S4:** Sample characteristics of neuroimaging dataset

|  | HIV+ (*N* = 11)  [n (%)] | HIV- Controls (*N* = 11)  [n (%)] | Test for significance | p-value |
| --- | --- | --- | --- | --- |
| Age | 39.91 (11.01) | 35.91 (7.46) | Z = -0.628 | 0.5302 |
| Sex: Female | 8 (72.73) | 6 (54.55) | $X^{2}$ = 0.196 | 0.6576 |
| Years of education | 10.18 (1.60) | 10.64 (1.63) | Z = 0.885 | 0.3759 |
| Currently taking ART | 4 (23.53) | – | – |  |
| Detectable HIV viral load | 6 (54.55) | – | – |  |
| HIV viral load (log transformed) | 8.52 (1.81) | – | – |  |
| Nadir CD4 count | 427.82 (184.77) | – | – |  |
| Nadir CD4 count <200 | 1 (9.09) | – | – |  |

For continuous variables (age, years of education, HIV viral load, nadir CD4), results are represented as: mean (standard deviation). For differences in age and years of education between groups the statistical test done was the Mann-Whitney for which the Z score is reported. For sex differences between groups the statistical test done was the Chi-squared test for which the $X^{2}$ value is reported. *Note:* ART use and detectability of viral load data missing for 4 participants, HIV viral load (log transformed) data missing for 1 participants. HIV, human immunodeficiency virus; HIV+, seropositive group; HIV-, seronegative control group; ART, antiretroviral therapy.

**Table S5**: Sample characteristics for comparisons between the whole sample (for NP data analysis) and the subgroup (for neuroimaging analysis) for local dataset

|  | | Whole sample (N = 39) | NI sample (N = 22) | Statistical tests |
| --- | --- | --- | --- | --- |
| All participants | | | | |
|  | *Age* | 39 (8.99) | 37.91 (9.41) | t = 0.442, df = 42.055,  p-value = 0.661 |
|  | *Sex: Female* | 24 (61.54) | 14 (63.64 ) | X^2^ = 0, df = 1,  p-value = 1 |
|  | *Years of education* | 10.54 (1.54) | 10.41 (1.59) | t = 0.308, df = 42.362,  p-value = 0.759 |
| HIV+ subgroups | | | | |
|  | *Currently on ART* | 6 (35.29) | 4 (36.36) | X^2^ = 0.394, df = 1,  p-value = 0.530 |
|  | *Detectable HIV viral loads* | 8 (47.06) | 6 (54.55) | X^2^ = 8.914 x 10^-33^, df = 1,  p-value = 1 |
|  | *Log HIV viral load* | 8.14 (1.91) | 8.52 (1.81) | t = -0.499, df = 20.22,  p-value = 0.623 |
|  | *Nadir CD4 count* | 439.75 (174.19) | 427.82 (184.77) | t = 0.169, df = 20.781,  p-value = 0.868 |
|  | *Nadir CD4 count <200* | 1 (5.88) | 1 (9.09) | X^2^ = 6.755 x 10^-31^, df = 1,  p-value = 1 |

For continuous variables (age, years of education, HIV viral load, nadir CD4), results are represented as: mean (standard deviation). For categorical variables (sex, currently on ART, detectable viral load, nadir CD4 count <200), results are shown as: n (%). For differences in continuous variables between groups the statistical test done was the t-test for which the t-value is reported. For differences in categorical variables between groups the statistical test done was the Chi-squared test for which the $X^{2}$ value is reported. *Note:* ART use and detectability of viral load data missing for 4 participants, HIV viral load (log transformed) data missing for 1 participants. HIV, human immunodeficiency virus; HIV+, seropositive group; HIV-, seronegative control group; ART, antiretroviral therapy.

**Table S6:** Selected Power et al. (2011) nodes from frontoparietal network

| ROI ID | MNI coordinates | | | Label |
| --- | --- | --- | --- | --- |
|  | X | Y | Z |  |
| 134 | -7 | -71 | 42 | l. precuneus |
| 175 | 48 | 25 | 27 | r. inferior frontal gyrus |
| 176 | -47 | 11 | 23 | l inferior frontal gyrus |
| 177 | -53 | -49 | 43 | l inferior parietal lobule |
| 186 | 47 | 10 | 33 | r. precentral gyrus |
| 187 | -41 | 6 | 33 | l. precentral gyrus |
| 188 | -42 | 38 | 21 | l. middle frontal gyrus |
| 193 | 32 | 14 | 56 | r. middle frontal gyrus |
| 194 | 37 | -65 | 40 | r. angular gyrus |
| 195 | -42 | -55 | 45 | l. angular gyrus |
| 197 | -34 | 55 | 4 | l middle frontal gyrus |
| 198 | -42 | 45 | -2 | l. middle orbital gyrus |
| 201 | -42 | 25 | 30 | l. inferior frontal gyrus |
| 202 | -3 | 26 | 44 | l. superior medial gyrus |
| 220 | -39 | 51 | 17 | l. middle frontal gyrus |

Images acquired in radiological orientation (left = right). ROI, region of interest.

**Section B:**

**NSLHAP Sample**

In recognition of the relatively small sample size of the local study, and to assess the robustness of the observed relationship between gait speed and neurocognitive function to differences in SES, our secondary aim was to replicate the observed associations between gait time and neurocognitive function in a larger US-based public domain dataset of older adults, provided as part of the National Social Life, Health, and Aging Project (NSHAP) (Waite *et al*, 2013). Specifically, data on gait and neurocognitive function collected during the 2^nd^ data collection phase of NSHAP was accessed through the National Archive of Computerized Data on Aging (NACDA). NSHAP is a US-based longitudinal, nationally representative study of older adults (61 to 91 years) with no known cognitive impairments. The second wave of NSHAP, conducted from 2010-2011, included assessments using a 3-meter timed gait task as well as the Chicago Cognitive Function Measure (CCFM) survey for a total sample of 3196 participants, data for which is included in this study.

**Neurocognitive testing**

The CCFM, as utilised within the NSHAP cohort, represents an adapted version of the Montreal Cognitive Assessment (MoCA), and was derived through a rigorous development process which concluded with pre-testing to evaluate the performance of the CCFM in the field before its successful incorporation into NSHAP, Wave 2 (Shega *et al*, 2014).

**Assessment of gait**

For the NSHAP cohort, participants were instructed to walk, at their normal walking speed, along a 3 metre path, with the average of the time taken to complete two administrations of this task employed as an indicator of gait speed.

**Statistical analysis and sample characteristics**

A total CCFM score was computed from the NSHAP dataset as the sum of all neurocognitive domain scores (total score range: 0 – 20). Z scores were generated for the total CCFM score as well as the average gait time, and included in the master dataset. From the 3100 participants who were able to successfully complete the walking task (out of a total sample of 3196), those who reported using walking aids (N = 597) and/or experiencing difficulty walking across a room (N = 452) were excluded from the dataset in an attempt to increase comparability with the local dataset. Additionally, participants with missing CCFM or average gait time estimates (N = 426), or with CCFM scores or average gait time estimates determined to be outliers (N = 3 & 17, respectively), using a Z score cut-off of 3.291, were identified and excluded. After applying these steps, a total of 1987 participated remained and were included in all subsequent analyses. Analyses in this study followed the recommendation by the NSHAP investigators to adjust for selection bias and non-response by including a weighting variable as a covariate in statistical models (see below).

Weighted multiple linear regression modelling frameworks were used to assess the strength of association between gait time and CCFM total scores, while adjusting for individual differences in age and sex. Additional models were run which included terms representing interaction effects between gait time and both sex and age. These models were also applied to Z-transformed gait time and CCFM total score estimates, thereby yielding a standardised model coefficient to facilitate comparisons with results from equivalent models for the local dataset. Of the 1987 participants from the NSHAP Wave 2 cohort included in the replication analysis, approximately half (49.72%) were female, with a mean (SD) age of 71.86 (6.81) years.

**NSLHAP weighted sample breakdown**

In Wave 2, the inclusion of romantic partners in households led to changes in within-household selection probabilities, necessitating the use of weights to provide unbiased estimates of population parameters. As described by O'Muircheartaigh et al. (2014), it is strongly recommended that the NSHAP data be weighted to adjust for selection bias and non-response by using the weighted variables already included in the public use dataset. The calculation of weights is rooted in Wave 1's base weight procedures. The base weight is derived from the inverse of the selection probability, accounting for household selection and individual sampling probabilities. These weights are further adjusted for changes in inclusion of participants in Wave 2.

Weighted variables in the Wave 2 dataset that were included in our statistical analysis:

Weight_adj – The primary weight for all Wave 2 analyses, adjusted for nonresponse.

Weight_sel – Adjusts for selection probabilities but excludes nonresponse adjustments.

We produced a new weighting variable which account for both nonresponse and selection probability by using the sum of Weight_adj and Weight_sel which was then normalized to sum to the sample size by dividing the combined weighting variable by its mean.

**Table S7:** Sample characteristics of NSHAP Wave 2 (N = 1987)

|  | Raw sample  [mean (SD)] | Weighted sample  [weighted mean (weighted SD)] |
| --- | --- | --- |
| Age | 71.86 (6.81) | 71.10 (6.66) |
| Sex: Female [n (%)] | 988 (49.72) | 983.90 (49.52) |
| Total CCFM Score ( /20) | 14.77 (3.18) | 15.02 (3.02) |
| Average gait time (seconds) | 4.45 (1.62) | 4.34 (1.54) |

For continuous variables (Age, Total CCFM Score, Average Gait Time), results are represented as: mean (standard deviation). For the categorical variable (sex), results are shown as: number of females (percentage). *Note:* CCFM, Chicago Cognitive Function Measure.

**Table S8:** Linear regression models for the effects of walk time and demographic variables on cognitive domain scores in the weighted NSHAP Wave II sample (N = 1987)

|  | | Model 1 | | | | | Model 2 | | | | |
| --- | --- | --- | --- | --- | --- | --- | --- | --- | --- | --- | --- |
|  |  | b | **SE** | **t-value** | **p-value** | **Adj.** $\boldsymbol{r}^{\boldsymbol{2}}$ | b | **SE** | **t-value** | **p-value** | **Adj.** $\boldsymbol{r}^{\boldsymbol{2}}$ |
|  |  |  |  |  |  |  |  |  |  |  |  |
| CCFM Total Score | |  |  |  |  | 0.089 |  |  |  |  | 0.092 |
|  | Walk time | -0.199 | 0.043 | -4.685 | **<0.001** |  | 1.202 | 0.481 | 2.498 | **0.013** |  |
|  | Age | -0.112 | 0.010 | -11.352 | **<0.001** |  | -0.0209 | 0.032 | -0.653 | 0.514 |  |
|  | Sex (♀) | 0.669 | 0.130 | 5.158 | **<0.001** |  | 0.595 | 0.389 | 1.530 | 0.126 |  |
|  | Walk time * Age | – | – | – | – |  | -0.020 | 0.007 | -2.982 | **0.003** |  |
|  | Walk time * Sex (♀) | – | – | – | – |  | 0.017 | 0.084 | 0.201 | 0.841 |  |
| Orientation score | |  |  |  |  | 0.010 |  |  |  |  | 0.009 |
|  | Walk time | -0.003 | 0.004 | -0.774 | 0.439 |  | -0.029 | 0.040 | -0.721 | 0.471 |  |
|  | Age | -0.001 | 0.001 | -1.690 | 0.091 |  | -0.003 | 0.003 | -1.118 | 0.264 |  |
|  | Sex (♀) | 0.046 | 0.011 | 4.313 | **<0.001** |  | 0.036 | 0.032 | 1.110 | 0.267 |  |
|  | Walk time * Age | – | – | – | – |  | <0.001 | <0.001 | 0.631 | 0.528 |  |
|  | Walk time * Sex (♀) | – | – | – | – |  | 0.002 | 0.007 | 0.346 | 0.730 |  |
| Naming score | |  |  |  |  | 0.018 |  |  |  |  | 0.017 |
|  | Walk time | -0.006 | 0.004 | -1.331 | 0.183 |  | -0.001 | 0.050 | -0.015 | 0.988 |  |
|  | Age | -0.006 | 0.001 | -5.637 | **<0.001** |  | -0.005 | 0.003 | -1.586 | 0.113 |  |
|  | Sex (♀) | -0.021 | 0.014 | -1.523 | 0.128 |  | -0.039 | 0.041 | -0.955 | 0.340 |  |
|  | Walk time * Age |  |  |  |  |  | <-0.001 | <0.001 | -0.149 | 0.882 |  |
|  | Walk time * Sex (♀) |  |  |  |  |  | 0.004 | 0.009 | 0.475 | 0.635 |  |
| Visuospatial score | |  |  |  |  | 0.018 |  |  |  |  | 0.020 |
|  | Walk time | -0.016 | 0.010 | -1.528 | 0.127 |  | 0.241 | 0.117 | 2.052 | **0.040** |  |
|  | Age | -0.014 | 0.002 | -5.713 | **<0.001** |  | 0.003 | 0.008 | 0.403 | 0.687 |  |
|  | Sex (♀) | -0.017 | 0.032 | -0.554 | 0.580 |  | -0.055 | 0.095 | -0.582 | 0.561 |  |
|  | Walk time * Age |  |  |  |  |  | -0.004 | 0.002 | -2.266 | **0.024** |  |
|  | Walk time * Sex (♀) |  |  |  |  |  | 0.009 | 0.021 | 0.421 | 0.674 |  |
| Executive function score | |  |  |  |  | 0.042 |  |  |  |  | 0.043 |
|  | Walk time | -0.010 | 0.007 | -1.492 | 0.136 |  | 0.112 | 0.077 | 1.458 | 0.145 |  |
|  | Age | -0.014 | 0.002 | -9.031 | **<0.001** |  | -0.006 | 0.005 | -1.204 | 0.229 |  |
|  | Sex (♀) | <-0.001 | 0.021 | -0.003 | 0.998 |  | -0.019 | 0.062 | -0.305 | 0.761 |  |
|  | Walk time * Age |  |  |  |  |  | -0.002 | 0.001 | -1.648 | 0.099 |  |
|  | Walk time * Sex (♀) |  |  |  |  |  | 0.004 | 0.014 | 0.323 | 0.747 |  |
| Attention score | |  |  |  |  | 0.032 |  |  |  |  | 0.032 |
|  | Walk time | -0.066 | 0.015 | -4.477 | **<0.001** |  | -0.139 | 0.167 | -0.830 | 0.406 |  |
|  | Age | -0.016 | 0.003 | -4.546 | **<0.001** |  | -0.018 | 0.011 | -1.667 | 0.096 |  |
|  | Sex (female) | -0.191 | 0.045 | -4.238 | **<0.001** |  | -0.388 | 0.135 | -2.873 | **0.004** |  |
|  | Walk time * Age |  |  |  |  |  | <0.001 | 0.002 | 0.295 | 0.768 |  |
|  | Walk time * Sex (♀) |  |  |  |  |  | 0.046 | 0.029 | 1.550 | 0.121 |  |
| Language score | |  |  |  |  | 0.038 |  |  |  |  | 0.040 |
|  | Walk time | -0.044 | 0.011 | -4.167 | **<0.001** |  | 0.258 | 0.119 | 2.163 | **0.031** |  |
|  | Age | -0.015 | 0.002 | -5.986 | **<0.001** |  | 0.005 | 0.008 | 0.610 | 0.542 |  |
|  | Sex (♀) | 0.142 | 0.032 | 4.429 | **<0.001** |  | 0.143 | 0.096 | 1.481 | 0.139 |  |
|  | Walk time * Age |  |  |  |  |  | -0.004 | 0.002 | -2.575 | **0.010** |  |
|  | Walk time * Sex (♀) |  |  |  |  |  | <-0.001 | 0.021 | -0.006 | 0.995 |  |
| Abstraction score | |  |  |  |  | 0.005 |  |  |  |  | 0.004 |
|  | Walk time | -0.015 | 0.007 | -2.156 | **0.031** |  | 0.085 | 0.080 | 1.071 | 0.284 |  |
|  | Age | -0.002 | 0.002 | -0.982 | 0.326 |  | 0.005 | 0.005 | 0.931 | 0.352 |  |
|  | Sex (♀) | -0.048 | 0.021 | -2.256 | **0.024** |  | -0.055 | 0.064 | -0.854 | 0.393 |  |
|  | Walk time * Age |  |  |  |  |  | -0.001 | 0.001 | -1.294 | 0.196 |  |
|  | Walk time * Sex (♀) |  |  |  |  |  | 0.002 | 0.014 | 0.108 | 0.914 |  |
| Short-term memory score | |  |  |  |  | 0.102 |  |  |  |  | 0.106 |
|  | Walk time | -0.040 | 0.021 | -1.845 | 0.065 |  | 0.674 | 0.242 | 2.780 | **0.005** |  |
|  | Age | -0.045 | 0.005 | -9.064 | **<0.001** |  | <-0.001 | 0.016 | -0.052 | 0.958 |  |
|  | Sex (♀) | 0.757 | 0.065 | 11.595 | **<0.001** |  | 0.972 | 0.196 | 4.965 | **<0.001** |  |
|  | Walk time * Age |  |  |  |  |  | -0.010 | 0.003 | -2.884 | **0.004** |  |
|  | Walk time * Sex (♀) |  |  |  |  |  | -0.050 | 0.043 | -1.165 | 0.244 |  |

Model 1 includes the independent effects of Walk time, Age and Sex (female) as predictor variables and the cognitive domain score as the outcome variable. Model 2 includes the interactive effects of Walk time-and-age and Walk time-and-sex as predictor variables and the cognitive domain score as the outcome variable. CCFM, Chicago Cognitive Function Measure; $\beta$, standardized coefficient; SE, standard error; Adj., adjusted; ♀: female. Bold indicates p < 0.05, unadjusted.

**Table S9:** Standardized linear regression models for the effects of walk time and demographic variables on cognitive domain scores in the weighted NSHAP Wave II sample (N = 1987)

|  | | Model 1 | | | | | Model 2 | | | | |
| --- | --- | --- | --- | --- | --- | --- | --- | --- | --- | --- | --- |
|  |  | $\beta$ | **SE** | **t-value** | **p-value** | **Adj.** $\boldsymbol{r}^{\boldsymbol{2}}$ | $\beta$ | **SE** | **t-value** | **p-value** | **Adj.** $\boldsymbol{r}^{\boldsymbol{2}}$ |
|  |  |  |  |  |  |  |  |  |  |  |  |
| CCFM Total Score | |  |  |  |  | 0.089 |  |  |  |  | 0.092 |
|  | Walk time | -0.102 | 0.022 | -4.685 | **<0.001** |  | -0.121 | 0.032 | -3.838 | **<0.001** |  |
|  | Age | -0.239 | 0.021 | -11.352 | **<0.001** |  | -0.236 | 0.021 | -11.189 | **<0.001** |  |
|  | Sex (♀) | 0.210 | 0.041 | 5.158 | **<0.001** |  | 0.212 | 0.041 | 5.165 | **<0.001** |  |
|  | Walk time * Age | – | – | – | – |  | -0.070 | 0.023 | -2.982 | **0.003** |  |
|  | Walk time * Sex (♀) | – | – | – | – |  | 0.009 | 0.043 | 0.201 | 0.841 |  |
| Orientation score | |  |  |  |  | 0.010 |  |  |  |  | 0.009 |
|  | Walk time | -0.018 | 0.023 | -0.774 | 0.439 |  | -0.023 | 0.034 | -0.681 | 0.496 |  |
|  | Age | -0.038 | 0.022 | -1.690 | 0.091 |  | -0.038 | 0.022 | -1.712 | 0.087 |  |
|  | Sex (♀) | 0.187 | 0.043 | 4.313 | **<0.001** |  | 0.188 | 0.044 | 4.326 | **<0.001** |  |
|  | Walk time * Age | – | – | – | – |  | 0.016 | 0.025 | 0.631 | 0.528 |  |
|  | Walk time * Sex (♀) | – | – | – | – |  | 0.016 | 0.046 | 0.346 | 0.730 |  |
| Naming score | |  |  |  |  | 0.018 |  |  |  |  | 0.017 |
|  | Walk time | -0.031 | 0.023 | -1.331 | 0.183 |  | -0.043 | 0.033 | -1.280 | 0.201 |  |
|  | Age | -0.126 | 0.022 | -5.637 | **<0.001** |  | -0.125 | 0.022 | -5.607 | **<0.001** |  |
|  | Sex (♀) | -0.066 | 0.043 | -1.523 | 0.128 |  | -0.064 | 0.043 | -1.485 | 0.138 |  |
|  | Walk time * Age |  |  |  |  |  | -0.004 | 0.025 | -0.149 | 0.882 |  |
|  | Walk time * Sex (♀) |  |  |  |  |  | 0.022 | 0.046 | 0.475 | 0.635 |  |
| Visuospatial score | |  |  |  |  | 0.018 |  |  |  |  | 0.020 |
|  | Walk time | -0.036 | 0.024 | -1.528 | 0.127 |  | -0.059 | 0.034 | -1.714 | 0.087 |  |
|  | Age | -0.130 | 0.023 | -5.713 | **<0.001** |  | -0.127 | 0.023 | -5.578 | **<0.001** |  |
|  | Sex (♀) | -0.024 | 0.044 | -0.554 | 0.580 |  | -0.023 | 0.044 | -0.525 | 0.599 |  |
|  | Walk time * Age |  |  |  |  |  | -0.057 | 0.025 | -2.266 | **0.024** |  |
|  | Walk time * Sex (♀) |  |  |  |  |  | 0.020 | 0.047 | 0.421 | 0.674 |  |
| Executive function score | |  |  |  |  | 0.042 |  |  |  |  | 0.043 |
|  | Walk time | -0.034 | 0.023 | -1.492 | 0.136 |  | -0.051 | 0.034 | -1.520 | 0.129 |  |
|  | Age | -0.202 | 0.022 | -9.031 | **<0.001** |  | -0.200 | 0.022 | -8.923 | **<0.001** |  |
|  | Sex (♀) | <0.001 | 0.043 | 0.003 | 0.998 |  | 0.001 | 0.043 | 0.024 | 0.981 |  |
|  | Walk time * Age |  |  |  |  |  | -0.041 | 0.025 | -1.648 | 0.099 |  |
|  | Walk time * Sex (♀) |  |  |  |  |  | 0.015 | 0.046 | 0.323 | 0.747 |  |
| Attention score | |  |  |  |  | 0.032 |  |  |  |  | 0.032 |
|  | Walk time | -0.100 | 0.022 | -4.477 | **<0.001** |  | -0.135 | 0.033 | -4.148 | **<0.001** |  |
|  | Age | -0.098 | 0.022 | -4.546 | **<0.001** |  | -0.098 | 0.022 | -4.520 | **<0.001** |  |
|  | Sex (female) | -0.178 | 0.042 | -4.238 | **<0.001** |  | -0.173 | 0.042 | -4.116 | **<0.001** |  |
|  | Walk time * Age |  |  |  |  |  | 0.007 | 0.024 | 0.295 | 0.768 |  |
|  | Walk time * Sex (♀) |  |  |  |  |  | 0.069 | 0.044 | 1.550 | 0.121 |  |
| Language score | |  |  |  |  | 0.038 |  |  |  |  | 0.040 |
|  | Walk time | -0.096 | 0.023 | -4.167 | **<0.001** |  | -0.109 | 0.033 | -3.265 | **0.001** |  |
|  | Age | -0.133 | 0.022 | -5.986 | **<0.001** |  | -0.130 | 0.022 | -5.846 | **<0.001** |  |
|  | Sex (♀) | 0.191 | 0.043 | 4.429 | **<0.001** |  | 0.191 | 0.043 | 4.419 | **<0.001** |  |
|  | Walk time * Age |  |  |  |  |  | -0.064 | 0.025 | -2.575 | **0.010** |  |
|  | Walk time * Sex (♀) |  |  |  |  |  | <-0.001 | 0.046 | -0.006 | 0.995 |  |
| Abstraction score | |  |  |  |  | 0.005 |  |  |  |  | 0.004 |
|  | Walk time | -0.051 | 0.024 | -2.156 | **0.031** |  | -0.061 | 0.034 | -1.762 | 0.078 |  |
|  | Age | -0.022 | 0.023 | -0.982 | 0.326 |  | -0.021 | 0.023 | -0.908 | 0.364 |  |
|  | Sex (♀) | -0.100 | 0.044 | -2.256 | **0.024** |  | -0.100 | 0.044 | -2.244 | **0.025** |  |
|  | Walk time * Age |  |  |  |  |  | -0.033 | 0.025 | -1.294 | 0.196 |  |
|  | Walk time * Sex (♀) |  |  |  |  |  | 0.005 | 0.047 | 0.108 | 0.914 |  |
| Short-term memory score | |  |  |  |  | 0.102 |  |  |  |  | 0.106 |
|  | Walk time | -0.041 | 0.022 | -1.845 | 0.065 |  | -0.028 | 0.032 | -0.880 | 0.379 |  |
|  | Age | -0.194 | 0.021 | -9.064 | **<0.001** |  | -0.191 | 0.021 | -8.937 | **<0.001** |  |
|  | Sex (♀) | 0.480 | 0.041 | 11.595 | **<0.001** |  | 0.476 | 0.041 | 11.499 | **<0.001** |  |
|  | Walk time * Age |  |  |  |  |  | -0.068 | 0.024 | -2.884 | **0.004** |  |
|  | Walk time * Sex (♀) |  |  |  |  |  | -0.051 | 0.044 | -1.165 | 0.244 |  |

Model 1 includes the independent effects of Walk time, Age and Sex (female) as predictor variables and the cognitive domain score as the outcome variable. Model 2 includes the interactive effects of Walk time-and-age and Walk time-and-sex as predictor variables and the cognitive domain score as the outcome variable. CCFM, Chicago Cognitive Function Measure; $\beta$, standardized coefficient; SE, standard error; Adj., adjusted; ♀: female. Bold indicates p < 0.05, unadjusted.

**Figure S1**. Neurocognitive performance as a function of gait time. Both gait time and neurocognitive performance scores are presented as standardized Z-scores on both axes. (A) Scatter plot illustrating relationship between gait time and global neurocognitive scores in local dataset, separated by HIV status. Regression lines are shown with shaded areas indicating 95% confidence intervals. *One participant from the HIV- group was excluded due to having missing data for the motor function domain and therefore for the Global neurocognitive score (N=38). (B) Hexagon plot illustrating relationship between gait time and total CCFM scores in the NSHAP dataset (N = 1987), graded by frequency (count). Regression line shown with shaded area indicating 95% confidence intervals.
